# Medical Humbug

**Published:** 1854-10

**Authors:** 


*Medical Humbugs.*

We presume our readers generally are aware that Dr. James McClintock, late Professor, &c., has sold himself to a New York druggist. We saw at the time numerous notices of his professional back-sliding; but it had almost escaped our memory till we were reminded of it this morning by a benevolent darkey, who thrust one of the extinguished professor's circulars into our hands. The following certificate indicates the reasons for the sale:

CERTIFICATE.

This is to certify that I have placed in the hands of Messrs. A. Cushman & Co., New York, the recipes of my "Family Medicines," with my full authority to prepare and offer them for popular use. They are such as I have been in the habit of recommending and prescribing for many years, and their composition is well known to hundreds of regular physicians who have been my students, and who are, many of them, now practising with distinguished success in all parts of the country. My inducements for this step are numerous: one of the most urgent is, that the demand for my remedies has become so large, through private report of their success, as to make their preparation too great a draft upon my time; another is the repeated solicitations of my friends and patients, in the belief that the general introduction of my remedies would be in many respects a public benefit; and fina ly, in securing the co-operation of Mr. Alex. Cushman, who is a thoroughly educated Chemist, and practical Apothecary of many years' experience, whereby I am enab ed to assure the public that they wi l be prepared in the best, purest, and most perfect form, regardless of expense.

JAMES McCLINTOCK, M. D.,

Late Professor of Anatomy and Surgery in the Philadelphia College of Medicine, and Acting Professor of Midwifery; one of the Consulting

Physicians of the Philadelphia Hospital, Blockley; late member of the National Medical Association; member of the Philadelphia Medical Society; member of the Medico Chirurgical College of Philadelphia; formerly President and Professor of Anatomy and Surgery in Castleton Medical College, Vermont; and also, late Professor of Anatomy and Physiology in Berkshire Medical Institution, Pittsfield, Mass., &c., &c., &c.

PHILADELPHIA, November, 1853.

☞The undersigned, in accepting the proprietorship of Dr. James Mc Clintock's Family Medicines, would respectfully state to the public, that in his proffesion as Dispensing Chemist, for nearly fifteeen years past, during which time he has been actively connected with the best pharmaceutical establishments in this country, he has had constant occasion to see and prepare the prescriptions of the most eminent physicians, among whom he would name Dr. Mott, Dr. Parker, Dr. Francis, Dr Cheeseman, Dr Reese, Dr. Whittaker, Dr. Schmidt, Dr Hosack, Dr. Kissam, and many others in the city of New York. The experience thus acquired enables him to discriminate with confidence between "legitimate medicines" combined scientifically, and the inaccurate mixtures of quackery and ignorance; wherein for want of proper knowledge in combining them, the best ingredients may neutralize and destroy each other. It is with pleasure, therefore, he testifies, that the prescriptions of Dr. James Mc Clintock are not only camposed of the most valuable medicinal agents employed by our best practitioners, but they are combined in such accurate proportions, in relation to their chemical and medical effects, as to secure the attainment of the utmost increase of their power and efficiency. In conclusion, he will only add for himself that he can refer with confidence to his past career, as his guarantee to the public, that these "great remedies" will be faithfully prepared in strict accordance with the prescriptions of their distinguished Originator.

ALEX. CUSHMAN,
*Graduate and Trustee of New York College of Pharmacy.*

The conditions of the sale are not given; but we have heard it intimated, that for the prescriptions and the use of his name he received fifty thousand dollars, or rather that for that sum he has sold himself, professional honor, reputation and influence, and has become a mere thing, the instrument of a pill maker and powder vender. Physicians are subject to temptations, like other men, and it can hardly be expected that of all who attain to eminence in the profession, there will be none who yield to the influence of wealth and ease. We claim with confidence, however, that such examples are fewer in our profession than in almost any other.

It is true there are all sorts of vampires hanging on to the skirts of medicine, ignorant pretenders, and shrewd charlatans, self-dubbed doctors accommodating themselves to every change of the popular sentiment for the purpose of winning the popular fee. In contrast with these are the practitioners of legitimate medicine, men who have patiently toiled year after year, slowly but surely solving one after another of the great mysteries of nature, listening to her voice as she speaks through insect, bird and beast, through flowers and trees, till at length with practiced ear and skilful hand, they are prepared to detect the discords of life's harp, attune its strings, and wake its wondrous harmonies. The study of truth in nature makes men better; it is for this reason that but few who have won an honorable admission to the ranks of our profession have disgraced it by prostituting themselves to quackery.

We had intended to say something in reference to the various isms and pathies of the day. We believe there is a reason for their existence, and that in their ultimate results they will add to the treasures of our science and the triumphs of our art No system either of religion, politics or medicine can gain to any great extent the confidence of the public unless it contain in itself something of truth, or be corrective of some wide-spread and popular abuse. Hydropathy and homœopathy will illustrate the idea. The first consists of the elevation of a subordinate truth in medicine to a central position. Water has long been known as a remedial agent; but not until quite recently was it ever regarded as a universal remedy. Its advocates have erected around it a system from which is carefully excluded every idea not consistent with the free and indiscriminate use of their grand panacea. The result of all this will be a thorough proving of water as a remedial agent; the indications for its use, and the good that can be accomplished by it, having been once thoroughly demonstrated, the errors of which the system has been constructed will be forgotten, and all of truth that remains will be re-absorbed into the body of legitimate medicine. The results of other partial systems will be precisely the same

Homœopathy, on the contrary, has had its origin in an abuse of active medicines, not by the profession alone, but by the people; and it is, perhaps, difficult to say in this case which is worse—the disease or the remedy. The first kills, the second allows the patient to die; the first introduces into the system disturbing forces, which may be right or may be wrong; the second withholds all correctives, and trusts alone to unaided nature. The mission of homœopathy is to show what nature can and cannot do. When this is once accomplished, Hannemann will be forgotten, while our science will be enriched by the numerous experiments of his followers.

It is thus that science progresses; its very errors, the germs from which its offshoots grow, are the occasions for the development of truth. J.

*To Members of the Illinois State Medical Society:*

As Treasurer of the Society I owe the members an apology. At the last Annual Meeting the Treasurer was directed to notify all members not present of the amount of the Annual Assessment, in time to receive payment of the same before the 1st of August. I prepared circulars accordingly, but soon found myself in the midst of a cholera epidemic, which required all my time at the bed-side of the sick, both day and night. The circulars were consequently not sent until so late a period that many did not receive them until after the time fixed for payment. The same circumstances which delayed the sending of the circulars has also delayed the publication of the transactions of the Society. The latter will be issued from the press soon. Still if any members have withheld their assessment on the supposition that it was too late, they can still forward it immediately and they will be in time to retain their names in the list of members.

N. S. DAVIS,
Treasurer of State Society.

*To Subscribers.* Some complaints have been made on account of the irregularlty and delay in the issue of the Journal during the last few months. When we entered upon editorial duty, three months since, we promised to have the evil remedied, but have found it impossible to do so until the present time. This number is issued during the first *half* of the month, and the next we trust will be fully up *to time*, viz.: on the first of the month.

One source of embarrassment which we have met with belongs to the subscribers. When the publisher has been urged to greater promptness, he has uniformly complained that many of the subscribers do not pay up, and when dunned they get angry. Now we think we are safe in saying, that in future, the Journal shall be issued with regularity and promptness, and we trust every subscriber will feel himself under obligations to pay equally regular and prompt. If so, all parties will get on easily and good-naturedly. D.

*Rush Medical College.*—The first Tuesday in this month was the time for commencing clinical instruction in the Hospital, and the opening of the dissecting rooms in the College. Already (Oct. 6th) quite a number of students have arrived in the city and have commenced their daily visits to the Mercy Hospital, which is very full of patients, both medical and surgical. A large proportion of the patients are laboring under the graver forms of fever and dysentery. The prospects are good for a large class in the College during the coming winter.

*Appointment.*—We see, by a recent notice, that Dr. S. B. Hunt has been appointed to the Chair of Anatomy in the medical department of the University of Buffalo. Dr. H. has been for some time connected with the *Buffalo Medical Journal.* He possesses energy and ability, and will do honor to the school with which he has become connected. J.

